# Liguzinediol potentiates the metabolic remodeling by activating the AMPK/SIRT3 pathway and represses Caspase-3/GSDME-mediated pyroptosis to ameliorate cardiotoxicity

**DOI:** 10.1186/s13020-024-00955-5

**Published:** 2024-06-14

**Authors:** Weijie Zhu, Naqi Lian, Jia Wang, Fengming Zhao, Bowen Liu, Jiaxing Sheng, Chenyan Zhang, Xuan Zhou, Wenbai Gao, Chen Xie, Haoyu Gu, Yuxin Zhang, Mianli Bian, Miao Jiang, Yu Li

**Affiliations:** 1 School of Medicine, Nanjing University of Chinese Medicine, Nanjing, 210023 China; 2 School of Senior Care Services and Management, Nanjing University of Chinese Medicine, Nanjing, 210023 China; 3 College of Acupuncture and Massage Health and Rehabilitation, Nanjing University of Chinese Medicine, Nanjing, 210023 China

**Keywords:** Liguzinediol, Cardiotoxicity, AMPK/SIRT3 pathway, Pyroptosis

## Abstract

**Background:**

Liguzinediol (Lig) has emerged as a promising candidate for mitigating Doxorubicin (DOX)-induced cardiotoxicity, a significant limitation in the clinical application of this widely used antineoplastic drug known for its efficacy. This study aimed to explore the effects and potential mechanisms underlying Lig’s protective role against DOX-induced cardiotoxicity.

**Methods:**

C57BL/6 mice were treated with DOX. Cardiac function changes were observed by echocardiography. Cardiac structure changes were observed by HE and Masson staining. Immunofluorescence was applied to visualize the cardiomyocyte apoptosis. Western blotting was used to detect the expression levels of AMP-activated protein kinase (AMPK), sirtuin 3 (SIRT3), Caspase-3 and gasdermin E N-terminal fragment (GSDME-N). These experiments confirmed that Lig had an ameliorative effect on DOX-induced cardiotoxicity in mice.

**Results:**

The results demonstrated that Lig effectively countered myocardial oxidative stress by modulating intracellular levels of reactive oxygen species (ROS), malondialdehyde (MDA), and superoxide dismutase (SOD). Lig reduced levels of creatine kinase (CK) and lactate dehydrogenase (LDH), while ameliorating histopathological changes and improving electrocardiogram profiles in vivo. Furthermore, the study revealed that Lig activated the AMPK/SIRT3 pathway, thereby enhancing mitochondrial function and attenuating myocardial cell apoptosis. In experiments with H9C2 cells treated with DOX, co-administration of the AMPK inhibitor compound C (CC) led to a significant increase in intracellular ROS levels. Lig intervention reversed these effects, along with the downregulation of GSDME-N, interleukin-1β (IL-1β), and interleukin-6 (IL-6), suggesting a potential role of Lig in mitigating Caspase-3/GSDME-mediated pyroptosis.

**Conclusion:**

The findings of this study suggest that Lig effectively alleviates DOX-induced cardiotoxicity through the activation of the AMPK/SIRT3 pathway, thereby presenting itself as a natural product with therapeutic potential for preventing DOX-associated cardiotoxicity. This novel approach may pave the way for the development of alternative strategies in the clinical management of DOX-induced cardiac complications.

**Supplementary Information:**

The online version contains supplementary material available at 10.1186/s13020-024-00955-5.

## Introduction

Doxorubicin (DOX)-based chemotherapy stands as a cornerstone in cancer treatment [1]. However, the significant adverse effects of DOX, particularly on the heart, pose substantial challenges to the health of cancer patients and survivors, limiting its clinical application [2]. Cardiotoxicity induced by DOX is well-established, showing both cumulative-dose-dependent acute and chronic forms, including aberrant arrhythmias, ventricular dysfunction, and heart failure [3]. Despite progress in understanding cellular pathways affected by anthracyclines, pinpointing the precise molecular origins of acute or late-stage cardiotoxicity remains elusive. Various mechanisms contribute to cardiotoxicity, such as oxidative stress, Ca^2+^ overload, DNA damage, mitochondrial dysfunction, and autophagic flux impairment [4, 5], culminating in cardiomyocyte death and exacerbating cardiac dysfunction [6]. It is generally thought that free radical-induced damage contributes to anthracycline-induced cardiotoxicity [4]. Free radical-induced damage is widely accepted as a key player in anthracycline-induced cardiotoxicity, with uncontrolled reactive oxygen species (ROS) generation identified as the primary cause of DOX-induced cellular injury and cardiac dysfunction [7]. The heart's susceptibility to free radical damage is heightened due to its less active antioxidant network and limited regenerative capability [8]. Consequently, identifying a novel therapeutic target to mitigate oxidative stress is crucial in addressing DOX-induced cardiotoxicity.

AMP-activated protein kinase (AMPK) serves as a pivotal sensor of cellular energy status across diverse cell types [5, 9]. Another regulator of energy and metabolism homeostasis is mitochondrial deacetylase SIRT3 [10]. Activation of AMPK not only elevates intracellular NAD^+^ concentrations but also activates SIRT3 [11, 12]. As a cellular energy sensor, AMPK is believed to enhance mitochondrial function and alleviate cell death, providing a potential avenue for intervention [13]. Sirtuins (SIRTs) are a highly conserved family in metabolism and stress responses [14]. SIRT3 mainly exists in mitochondria and is a major mitochondrial deacetylase, which plays a key role in regulating mitochondrial energy and metabolic homeostasis. Pyroptosis, distinguished by unique morphological and biochemical characteristics, plays a role in DOX-induced cardiotoxicity [15]. Pyroptosis is defined as gasdermin-mediated programmed cell death characterized by inflammatory stimulation, cell membrane perforation, and release of mature IL-1β [16]. It is well known that pyroptosis is mainly mediated by caspase, Gasdermin E (GSDME), is cleaved specifically by caspase-3 to produce N-terminal fragments that ultimately lead to pyroptosis. Caspase-3 serves as a mediator not only for apoptosis but also for pyroptosis via GSDME cleavage [17]. In addition, the disturbance of energy metabolism will increase the ROS of cells, resulting in mitochondrial damage and ultimately apoptosis.

Previous studies determined that restraining oxidative stress and apoptosis were sufficient to ameliorate DOX-related cardiac damage and dysfunction [6, 7, 13]. The accumulation of DOX triggers cardiotoxicity, damaging cardiomyocytes. This damage disrupts the energy metabolism of heart muscle cells, leading to ATP depletion, inflammatory factor release, and cell membrane destruction, culminating in pyroptosis. Concurrently, disturbed energy metabolism boosts ROS production. The surplus ROS causes oxidative harm to mitochondria, reducing mitochondrial membrane permeability and exacerbating internal mitochondrial environment imbalances, ultimately inducing pyroptosis or apoptosis. Despite the recognized importance of restraining oxidative stress and apoptosis in ameliorating DOX-related cardiac damage, the precise molecular mechanism underlying the AMPK/SIRT3 pathway in inducing mitochondrial dysfunction and driving cardiomyocyte cell pyroptosis remains unexplored. Given the limited therapeutic options for cardiotoxicity, there is a critical need for novel approaches to address this challenge.

Liguzinediol (Lig), a derivative derived from ligustrazine found in Ligusticum wallichii Franch, has undergone structural modifications to capitalize on the active compound's properties. Previous studies from our research team have unveiled Lig’s ability to induce myotonic effects on normal isolated rat hearts, along with its potential to restore impaired cardiac contractility [18]. Notably, Lig has demonstrated efficacy in reducing myocardial cell apoptosis in stress-induced heart failure and promoting autophagy in salvaged cardiomyocytes [19, 20]. Building on these findings, our current investigation seeks to establish a heart failure model induced by DOX to scrutinize Lig’s protective effects against oxidative stress. Our goal is to unravel whether Lig achieves this protective action by modulating the AMPK/SIRT3 pathway, thereby alleviating caspase-3/GSDME-mediated pyroptosis triggered by mitochondrial dysfunction.

## Materials and methods

### Drugs and reagents

Lig was generously provided by Professor Li Wei from Nanjing University of Chinese Medicine. DOX was sourced from Yuanye in Shanghai, China, while Trimetazidine (TMZ) was obtained from RAHWN, also in Shanghai, China. Compound C (CC), a selective AMPK inhibitor, was procured from MCE in New Jersey, USA. Antibodies utilized in this study include anti-phospho-AMPK (p-AMPK, Thr172, #2535) and anti-cleaved-caspase-3 (#9664) from Cell Signaling Technology in Beverly, USA; anti-caspase-3 (BS1518), anti-β-actin (BS6007M), anti-IL-1β (BS3506) from Bioworld in Minnesota, USA; anti-AMPK (YT0215) from Immunoway in Beijing, China; and anti-Bcl-2 (68103-1-lg), anti-Bax (60267-1-lg), anti-IL-6 (23457-1-AP), anti-SIRT3 (10099-1-AP) from Proteintech in Wuhan, China. Additionally, goat anti-rabbit and goat anti-mouse secondary antibodies were obtained from Bioworld in Minnesota, USA. The Cell Counting Kit-8 (CCK-8) was purchased from Vazyme in Nanjing, Jiangsu, China. Kits for measuring malondialdehyde (MDA) concentration, superoxide dismutase (SOD) activity, lactic dehydrogenase (LDH) concentration, and adenosine triphosphate (ATP) content were sourced from Nanjing Jiancheng Bioengineering Institute in Nanjing, Jiangsu, China. ROS and JC-1 kits were purchased from Beyotime in Shanghai, China, and the PI dye solution was obtained from Abbkine in California, USA.

### Cell culture and siRNA transfection

H9C2 cells were procured from Shanghai Baili Biotechnology Co., Ltd. and were cultured in Dulbecco’s modified Eagle’s medium (DMEM, Gibco, USA) supplemented with 10% fetal bovine serum (FBS, Corning) and 1% penicillin–streptomycin (PS, Gibco) within a CO_2_ incubator set at 5% CO_2_ and 37 ℃ with saturated humidity. AMPK siRNA and GSDME siRNA were obtained from KeyGEN Biotech (Nanjing, Jiangsu, China). The H9C2 cells were seeded onto 6-well plates in 2 mL of antibiotic-free normal growth medium with FBS at a ratio of 1 × 10^5^ cells per well. When the cells reached 60–70% confluence, targeted siRNA duplex (100 pmol/L) was transfected using Lipofectamine 2000 (Invitrogen). Cells were then harvested 24 h post-transfection for subsequent experiments.

### Animal study

All experimental procedures strictly adhered to the PR China Legislation Regarding the Use and Care of Laboratory Animals. Ethical approval for all animal experiments was obtained from the Animal Care and Use Committee of Nanjing University of Chinese Medicine. Rats and mice for the experiments were procured from Hangzhou Medical College. The animals were group-housed in a controlled environment with a 12 h light/dark cycle, a temperature set at 25 ± 2 °C, and provided with free access to water and food. To ensure acclimatization, the animals were given 1 week before the commencement of the experiments.

### In vivo cardiotoxicity model

C57BL/6 mice, aged 5 weeks and weighing between 18 and 22 g, were utilized to establish chronic cardiotoxicity models through a 5-week low-dose treatment protocol. The mice were randomly allocated into six groups, each comprising 10 individuals: (i) control group; (ii) DOX treatment group; (iii) DOX and Lig cotreatment group; (iv) DOX and trimetazidine (TMZ) cotreatment group; (v) DOX and AMPK inhibitor (CC) cotreatment group; (vi) DOX, Lig, and AMPK inhibitor cotreatment group. The chronic cardiotoxicity model was induced by administering DOX at a dose of 5 mg/kg per week, dissolved in saline, through intraperitoneal injection over a span of 5 weeks. Consequently, the total cumulative dose of DOX administered was 25 mg/kg [21]. Then, Lig (20 mg/kg/day) [19] and CC (20 mg/kg/day) [22] were injected intraperitoneally for 4 weeks after DOX modeling. Lig, DOX, and compound C (CC) were initially dissolved in dimethyl sulfoxide (DMSO) and subsequently diluted with saline until reaching a final concentration of 1% DMSO (vehicle). Subsequently, cardiac function was assessed through echocardiography. Blood samples were collected from the orbital region to measure biochemical parameters using an automatic biochemical analyzer. Upon completion of the experiments, the mice were euthanized with sodium pentobarbital (200 mg/kg, administered via intraperitoneal injection). Subsequently, the hearts were excised and stored either at − 80 °C or fixed in a 4% paraformaldehyde buffer for further experimental procedures.

### Establishment of myocardial infarction rats model

Eight-week-old male Sprague–Dawley rats were obtained from the Institute of Biomedical Sciences at Nanjing University. After a week of acclimatization with normal feeding, the rats were anesthetized with 3% pentobarbital sodium at a dose of 40 mg/kg and intubated with an automatic respirator. In the model group, the anterior descending branch of the left coronary artery was ligated using a suture needle, and the chest was promptly closed (n = 6). The sham operation group underwent the same surgical procedure but without ligation of the coronary artery. Successful coronary artery ligation was confirmed by electrocardiogram assessment.

### Analysis of cell viability

Cell viability was assessed using the CCK-8 kit following the manufacturer's instructions. H9C2 cells (1 × 10^4^) were seeded in 96-well plates, with five parallel replicates prepared for each treatment. The cells were treated with various concentrations of Lig (0–1000 μM) for 24 h, and the control group received treatment with DMSO (0.1%). After the 24 h incubation period, cell viabilities were determined using the CCK-8 assay. Specifically, CCK-8 (20 μL) was added to each well containing 200 μL of medium and incubated at 37 ℃ for 3 h. Optical density (OD) values were measured at 450 nm using an enzyme-labeled instrument (BioTek-Elx800, Vermont, USA), and the ratio of OD values between experimental and control wells was utilized to indicate cell viability.

### Echocardiography

Upon completion of the Lig treatment, mice were gently anesthetized with 1.5% isoflurane until their heart rates stabilized at a depth of 400–500 beats per minute. Echocardiography assessments were conducted using the VEVO 3100 high-frequency color ultrasound system from VisualSonics in Toronto, Canada. Left ventricular ejection fraction (LVEF) and fraction shortening (FS) were measured or calculated based on the echocardiographic data, employing the described procedures.

### Analysis of ATP, LDH, SOD and MDA

The quantification of ATP, LDH, SOD, and MDA levels in H9C2 cells and heart tissue was carried out using kits obtained from Jiancheng Bioengineering Institute in Nanjing, China. The experimental procedures were conducted in accordance with the provided protocol from the manufacturer. The kits facilitated the measurement of these specific parameters, providing a comprehensive analysis of cellular and tissue responses.

### Western blotting assay

For both cells and heart tissues, total protein samples were homogenized using RIPA lysis buffer containing protease and phosphatase inhibitors. The protein concentrations were determined using a BCA Protein Assay Kit. Subsequently, the protein samples from each group were separated by SDS-PAGE and transferred to a PVDF membrane. The membrane was then incubated with 5% fat-free milk at room temperature for 2 h, followed by overnight incubation with primary antibodies against AMPK, p-AMPK, SIRT3, Bax, Bcl-2, IL-6, IL-1β, Cleaved-caspase-3, Caspase-3, GSDME-N, and β-actin at 4 ℃. After washing in TBST, the membrane was incubated with the corresponding secondary antibody for 2 h at room temperature. Fluorescence signals were detected using the BioRad imaging system (BioRad, Hercules, USA) and quantified using Image Lab Software (BioRad, Hercules, USA).

### Measurement of intracellular ROS

H9C2 cells were seeded in 6-well culture plates at a density of 5 × 10^4^ cells/mL and pre-treated with Lig for 24 h before exposure to DOX for an additional 24 h. Subsequently, DCFH-DA (10.0 μM, 1.5 mL) was added to the wells and allowed to induce for 25 min at 37 °C. The samples were then observed using fluorescence microscopy (Observer.Z1, Zeiss, Germany) at a magnification of 200×. This process allowed for the visualization and assessment of fluorescence intensity indicative of intracellular ROS levels.

### Hoechst staining

Cell slivers were positioned in a 24-well plate, and H9C2 cells were inoculated into the wells. Following the completion of the culture, staining was carried out using the Apoptosis Assay Kit from Beyotime Institute of Biotechnology, following the manufacturer’s instructions. The cells were stained with 10 ng/mL Hoechst 33342 for 5 min at 24 °C in the dark. Subsequently, the nuclei of apoptotic cells were visualized and assessed using a fluorescence microscope (Observer.Z1, Zeiss, Germany). This process facilitated the identification and observation of apoptotic cell nuclei.

### Propidium iodide (PI) staining

The cell sliver was positioned in a 24-well plate, and H9C2 cells were inoculated into the wells. Following the completion of the culture, staining was conducted using the kits from Beyotime Institute of Biotechnology. Cells were stained with 10 ng/mL PI for 20 min at 0 °C in the dark. Subsequently, the nuclei of cells undergoing necrosis were visualized and assessed using a fluorescence microscope (Observer.Z1, Zeiss, Germany). This process facilitated the identification and observation of cells undergoing necrosis based on PI staining.

### Mitochondrial membrane potential (MMP) detection

MMP levels in H9C2 cells were assessed using the detection kits following the provided instructions. H9C2 cells were fixed with 4% paraformaldehyde and then exposed to the appropriate MMP probe. Subsequently, MMP fluorescence was observed using fluorescence microscopy (Observer.Z1, Zeiss, Germany). This approach allowed for the visualization and quantification of changes in MMP based on the fluorescent signals emitted by the probe.

### Analysis of creatine kinase-MB (CK-MB)

To assess the level of myocardial injury, the content of CK-MB in the serum was measured. The detection was carried out following the instructions provided with the test kit. The specific procedures outlined in the kit's instructions were followed to accurately quantify the level of CK-MB in the serum, providing valuable information about myocardial health and potential injury.

### Hematoxylin and eosin (HE) staining

The heart tissue was fixed using 4% paraformaldehyde, dehydrated with ethanol, cleared with xylene, and subsequently embedded in paraffin. The paraffin-embedded heart tissue was then sectioned into 5 μm slices using a microtome. After dewaxing, the sections were stained with HE. The stained sections were observed and photographed under an optical microscope. This histological approach allowed for the examination of tissue architecture and cellular details in the heart tissue.

### Masson staining

The fixed heart tissue underwent deparaffinization with ethanol and xylene, followed by paraffin embedding and slicing into 5 μm sections. After dewaxing, the sections were stained using the Masson kit following the recommended procedures. Subsequently, the stained sections were observed and photographed under a light microscope. This staining technique, commonly used for collagen visualization, provides insights into tissue fibrosis and structural changes.

### Analysis of immunofluorescence

The prepared tissue slices underwent dewaxing and were subsequently permeabilized with 2% Triton for 20 min. Following this, the slices were incubated with 5% BSA at room temperature for 1 h. Primary antibodies were then added, and the slices were kept at 4 ℃ overnight. On the following day, PBS was used to remove unbound antibodies, and the slices were washed three times. Fluorescent secondary antibodies were added and incubated at room temperature for 2 h away from light. Subsequently, DAPI was added for 3 min to stain the cell nuclei. Finally, the slices were observed and photographed under a fluorescence microscope (Observer.Z1, Zeiss, Germany). This method enabled the visualization of specific cellular components and proteins labeled with fluorescent markers.

### Statistical analysis

All data were analyzed using GraphPad Prism 9.0 software (GraphPad Software Co., Ltd, USA) and presented as mean ± SD. Statistical analyses, including univariate analysis of variance (ANOVA) and LSD (Least Significant Difference) analysis, were performed on the experimental results using GraphPad Prism 9.0 software. A significance level of *P* < 0.05 was used to identify statistically significant differences.

## Results

### Lig protects against DOX-induced heart failure and alleviates oxidative stress in vivo

**Fig. 1:**
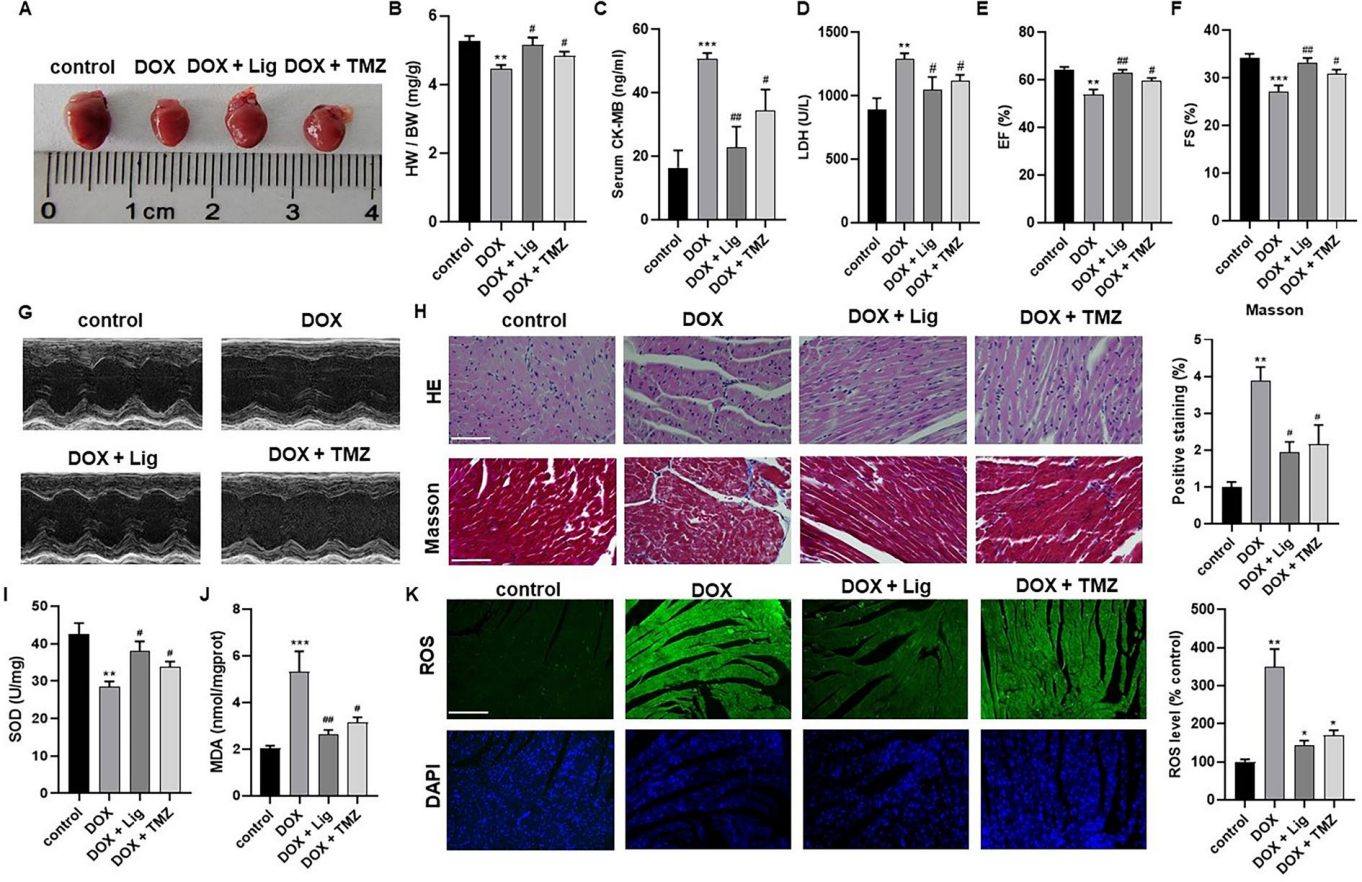
Lig improves DOX-induced cardiac dysfunction in mice. **A**, **B** The gross heart pictures and the HW/BW ratio of mice (n = 10). **C**, **D** Effects of Lig on serum levels of CK-MB and LDH in mice caused by DOX (n = 6). **E**–**G** The LVEF and LVFS were measured by echocardiography in each group (n = 6). **H** HE and Masson staining images of heart tissues in mice (n = 3). **I**, **J** MDA and SOD levels in mice caused by DOX (n = 6). **K** Effects of Lig on heart levels of ROS caused by DOX (n = 3). Data are presented as the mean ± SD. **p < 0.01, ***p < 0.001 compared with control group; ^*#*^*p* < 0.05, ^##^*p* < 0.01 compared with model group

DOX-induced cardiotoxicity is a classical chronic cardiotoxic model [2, 3]. The mice received a low dose of DOX (5 mg/kg/week) intraperitoneally for 5 weeks. Based on the therapeutic outcomes, a dose of Lig (20 mg/kg/day) and TMZ (10 mg/kg/day) were selected for the treatment of DOX-induced cardiotoxicity in mice for 1 month. TMZ is used as a clinical treatment for cardiac diseases by improving myocardial energy homeostasis and cardiac systolic-diastolic function; therefore, we served as a positive control drug [23]. After treatment, efforts were made to assess the anti-atrophy effect of Lig. The results revealed that the heart weight-to-body weight ratio decreased in DOX-treated mice compared to saline-treated mice, while it increased in Lig-treated mice (Fig. 1A and B). Furthermore, the cardiotoxicity of DOX was evaluated using biochemical markers associated with heart failure. The data showed a significant increase in the concentrations of CK-MB and LDH after DOX treatment. However, following treatment with Lig and TMZ, the concentrations of CK-MB and LDH decreased, with noteworthy emphasis on Lig's superior therapeutic effect compared to TMZ (Fig. 1C and D). Cardiac function was assessed by echocardiography, focusing on ejection fraction (EF) and shortening fraction (FS). Compared with mice treated with normal saline, EF and FS in DOX-treated mice were significantly reduced, indicating that DOX-induced cardiac dysfunction in mice. Treatment with Lig and TMZ resulted in significantly higher EF and FS compared to the DOX group (Fig. 1E–G). Additionally, HE staining and Masson trichrome staining revealed improvements in myocardial inflammatory infiltration, expansion of intercellular spaces, and fibrosis in DOX-treated mice after treatment with Lig and TMZ (Fig. 1H). These findings suggest that Lig can ameliorate cardiac dysfunction in DOX-induced mice and improve structural changes associated with heart failure. In our previous study, we have also confirmed that Lig has positive muscle strength in rats with heart failure [19]. Subsequently, we conducted tests to verify this phenomenon in heart failure rats, where the left anterior descending coronary artery was ligated, followed by 8 weeks of Lig gavage (Fig. s1A). In hemodynamic assessments, we observed a gradual improvement in left ventricular systolic pressure (LVSP), left ventricular end-diastolic pressure (LVEDP), +dp/dt, and −dp/dt with increasing Lig dosage (Fig. s1B–E). Further tests revealed a gradual decrease in the levels of nitric oxide (NO) and free fatty acids (FFA) under Lig treatment (Fig. s1F and G). These preliminary results suggest that Lig plays a certain role in the treatment of heart failure.

Considering that mitochondrial oxidative stress plays a crucial role in the development of cardiac dysfunction under various pathological conditions [24]. The levels of ROS, MDA, and SOD in the myocardial tissue of mice were assessed. ROS tissue fluorescence analysis indicated an increase in ROS expression after DOX treatment. However, the ROS content in the heart decreased after treatment with Lig and TMZ. Regarding MDA and SOD, MDA content increased, and SOD activity decreased in the hearts of the DOX group, while MDA content decreased, and SOD activity increased under the treatment of Lig (Fig. 1I and J). Additionally, oxidative damage in rats was evaluated based on MDA, SOD, and ATP contents, and the results were consistent with the DOX-induced chronic cardiotoxic model in mice (Fig. s1H–J). ROS has the potential to induce apoptosis by affecting mitochondrial metabolism [25]. Western blotting analysis revealed that Lig treatment decreased cardiomyocyte apoptosis by reducing the Bax protein level and increasing the Bcl-2 protein level (Fig. s2A). Furthermore, immunofluorescence analysis demonstrated that apoptosis-inducing factor (AIF) was more expressed in the DOX group, but after treatment with Lig and TMZ, AIF expression decreased, with Lig exhibiting a better therapeutic effect than TMZ (Fig. s2B). These results collectively suggest that Lig can counteract oxidative stress damage and apoptosis induced by cardiotoxicity in vivo.

### Lig activates AMPK/SIRT3 signaling and alleviates pyroptosis mediated by Caspase-3/GSDME in DOX-induced cardiac toxicity

**Fig. 2:**
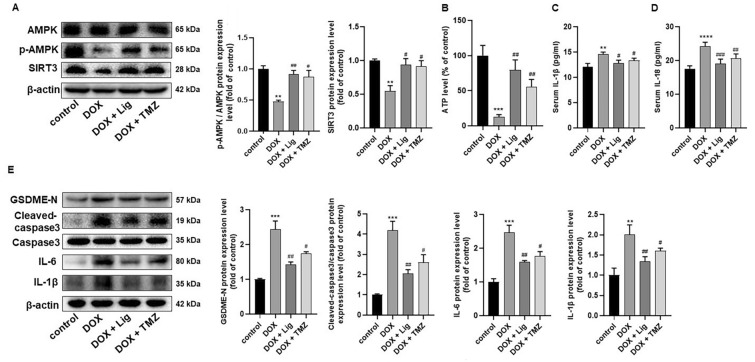
Lig improves DOX-induced cardiac toxicity in mice by activating AMPK/SIRT1 signaling and alleviating pyroptosis mediated by caspase-3/GSDME. **A** Representative western blotting images and statistical results of p-AMPK, AMPK and SIRT3 in mice (n = 3). **B** Effects of Lig on heart levels of ATP in mice caused by DOX (n = 5). **C**, **D** The serum levels of IL-1β and IL-18 of mice measured by ELISA. **E** The expression of GSDME-N, cleaved-caspase-3, caspase-3, IL-1β and IL-6 (n = 3). Data are presented as the mean ± SD. **p < 0.01, ***p < 0.001, ****p < 0.0001 compared with control group; ^#^*p* < 0.05, ^##^*p* < 0.01, ^###^*p* < 0.001 compared with model group

In recent years, numerous studies have highlighted that DOX-induced myocardial injury involves multiple biological processes, including oxidative stress, lipid peroxidation, DNA damage, mitochondrial injury, apoptosis, and autophagy. Among these processes, AMPK has emerged as a pivotal regulator of endogenous defense mechanisms against various pathological processes in the heart [26, 27]. To confirm whether AMPK and SIRT3 signaling are involved in Lig’s protection against DOX-induced myocardial injury in vivo, Fig. 2A demonstrates that Lig treatment significantly increased p-AMPK levels and promoted SIRT3 protein expression. As AMPK is a key regulator of biological energy metabolism, consistent with the aforementioned results, cardiac ATP content decreased in the DOX group and significantly increased after Lig treatment compared with the DOX group (Fig. 2B). In heart failure rats, we also observed significant increases in p-AMPK and SIRT3 under Lig treatment (Fig. S1K). Hence, Lig may represent an effective prevention and treatment method by activating the AMPK/SIRT3 signaling pathway against DOX-induced cardiotoxicity.

Activation of AMPK/SIRT3 signaling has been reported to induce pyroptosis [28]. Pyroptosis is recognized as a programmed cell death regulated by inflammation. Serum levels of inflammatory factors IL-1β and IL-18 increased in the DOX group, whereas they decreased significantly after treatment with Lig (Fig. 2C and D). GSDME, specifically cleaved by caspase-3, releases its N-terminal pore-forming domain under certain apoptotic stimuli, leading to pyroptotic death. In the DOX group, GSDME and Cleaved-caspase-3 protein levels increased; however, it was reversed after Lig treatment (Fig. 2E). Similarly, in heart failure rats, GSDME and Cleaved-caspase-3 protein levels were significantly reduced after treatment with Lig (Fig. S1K). The expression of related inflammatory factors IL-1β and IL-6 also decreased after Lig treatment compared with those in the DOX group (Fig. 2E). NF-κB has pro-inflammatory effects and is associated with pyroptosis. Western blot results showed that NF-κB activation increased in the DOX group and decreased NF-κB activation after Lig intervention (Fig. S2A). Collectively, these results indicate that Lig significantly improves AMPK/SIRT3 signaling and reduces pyroptosis in vivo.

### Lig improves DOX-induced H9C2 cell viability and decreases mitochondrial oxidative stress by activating AMPK/SIRT3 signaling

**Fig. 3:**
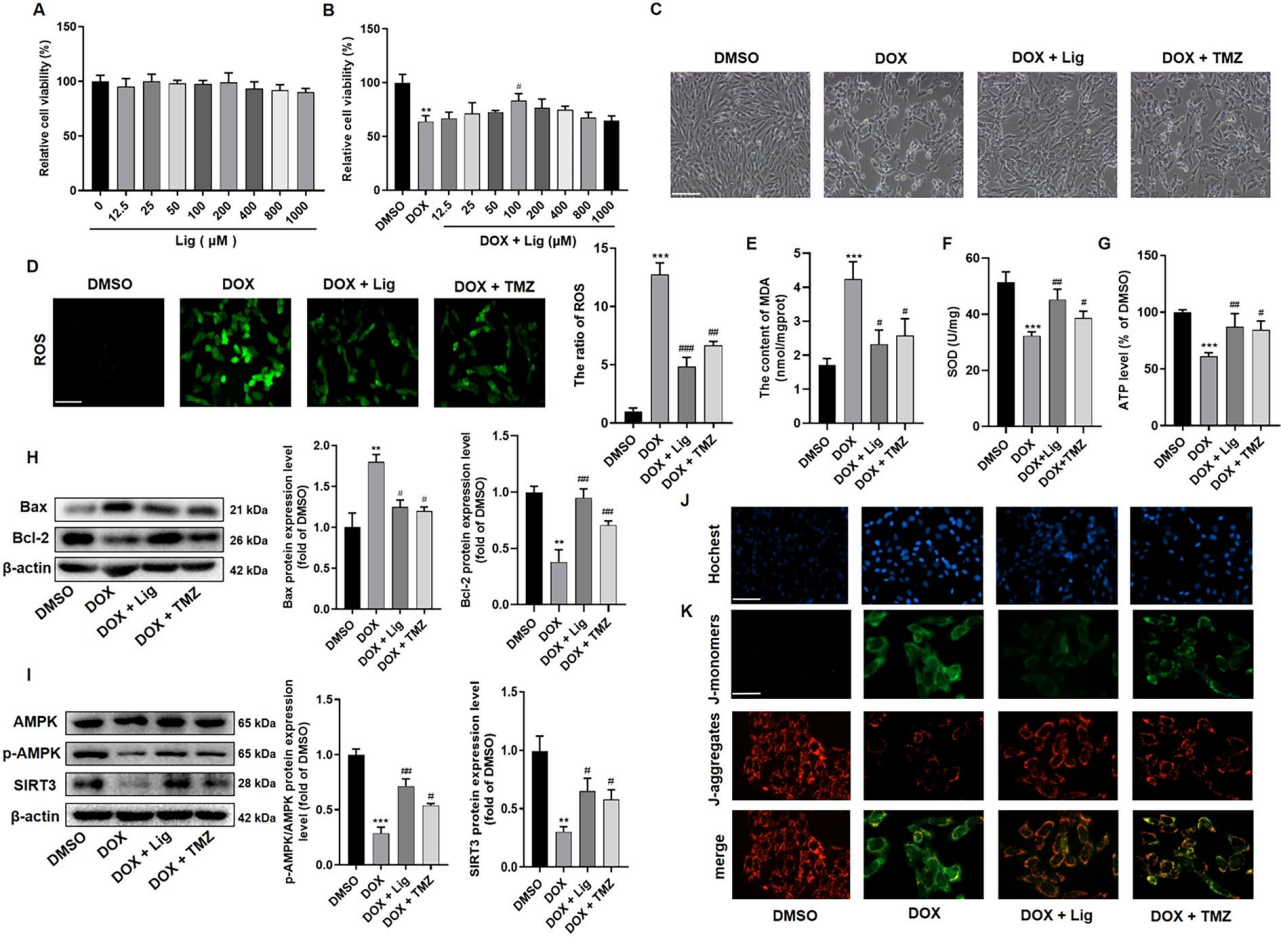
Lig decreased DOX-induced H9C2 cells viability inhibition, mitochondrial oxidative stress, and cell apoptosis by improving AMPK/SIRT3 signaling. **A** Cytotoxicity of Lig on H9C2 cells (n = 5). **B** Effects of Lig on the cell viability of H9C2 cells induced by DOX (n = 5). **C** The cellular morphology of H9C2 cells in bright image (n = 6). Scale bar represents 100 µm. **D** Intracellular ROS level in H9C2 cells (n = 4). Scale bar represents 50 µm. **E**–**G** Intracellular MDA, SOD and ATP level in H9C2 cells (n = 3). **H**, **I** The expression of Bax, Bcl-2, p-AMPK, AMPK and SIRT3 in H9C2 cells by western blotting assays (n = 3). **J** Representative images of Hochest staining (n = 6). Scale bar represents 50 µm. **K** The level of MMP was measured by JC-1 assay kit (n = 6). Scale bar represents 25 µm. Data are presented as the mean ± SD. **p < 0.01, ***p < 0.001 compared with control group; ^#^*p* < 0.05, ^##^*p* < 0.01, ^###^*p* < 0.001 compared with model group

Indeed, DOX can induce both acute and chronic cardiotoxicity, manifesting as various cardiovascular complications. These complications include tachycardia, arrhythmia, hypotension, transient depression of left ventricular function, and in severe cases, refractory late-onset cardiomyopathy [29]. To investigate whether Lig has a protective effect against DOX-induced cardiotoxicity in vitro, we initially examined its impact on cell viability. As depicted in Fig. 3A, compared with the control group, the viability of H9C2 cells treated with various concentrations of Lig did not exhibit significant changes within 24 h. This indicates that Lig did not exert significant toxicity on H9C2 cells at the experimental concentrations. In comparison to the DOX (3 μM) treatment group, different doses of Lig were found to enhance the viability of H9C2 cells, with Lig (100 μM) significantly improving cell viability and morphological damage in H9C2 cells (Fig. 3B and C). Subsequently, intracellular ROS production was analyzed based on ROS-mediated conversion of non-fluorescent 2′,7′-DCFH-DA to fluorescent DCFH, which exhibited enhanced fluorescence following the production of reactive metabolites [24]. In H9C2 cells treated with DOX, there was a significant increase in ROS production, and Lig treatment demonstrated a more effective inhibition of ROS production compared to TMZ-treated H9C2 cells (Fig. 3D). Similarly, we detected cellular ROS production by flow cytometry with the same results as in Fig. 3D (Fig. S3A). Additionally, MDA content increased, and SOD activity decreased in DOX-treated cells, which were reversed after Lig treatment (Fig. 3E and F). ATP levels exhibited a similar trend in cells (Fig. 3G). Similarly, levels of Bax and Bcl-2 were detected by western blotting, revealing an increase in Bax protein and a decrease in Bcl-2 protein levels in DOX-induced H9C2 cells. After Lig treatment, the Bax protein level decreased, and the Bcl-2 protein level increased (Fig. 3H). Hoechst can penetrate the cell membrane and bind to DNA, and in apoptotic cells, Hoechst-stained nuclei appear in a more pronounced blue light. Hoechst staining further confirmed that apoptosis of H9C2 cells treated with DOX was evident, and this apoptosis significantly decreased after Lig incubation (Fig. 3J and Fig. S3B). Collectively, these data suggest that Lig improves DOX-induced viability and reduces oxidative stress in H9C2 cells.

Consistent with the findings in vivo, which demonstrated that Lig stimulates AMPK/SIRT3 signaling and mitigates DOX-induced cardiac toxicity damage, we also assessed the protein levels of AMPK and SIRT3 in H9C2 cells. The results revealed that the protein expression levels were consistent with those observed in mice (Fig. 3I). The fundamental role of AMPK is to maintain mitochondrial health, and SIRT3 exerts regulatory effects on the structure and function of mitochondria. Mitochondrial dysfunction can lead to a dissipation of MMP and an increase in ROS. Therefore, we utilized the JC-1 assay and the data indicated that the membrane potential of H9C2 cells treated with Lig and TMZ significantly increased, with the membrane potential of Lig-treated cells higher than that of the TMZ group (Fig. 3K). These results suggest that Lig plays a myocardial protective role against DOX-induced cytotoxicity by activating AMPK and SIRT3 signaling pathways to improve mitochondrial function.

### Lig inhibits pyroptosis-associated proteins caspase-3 and GSDME in DOX-treated H9C2 cells

**Fig. 4:**
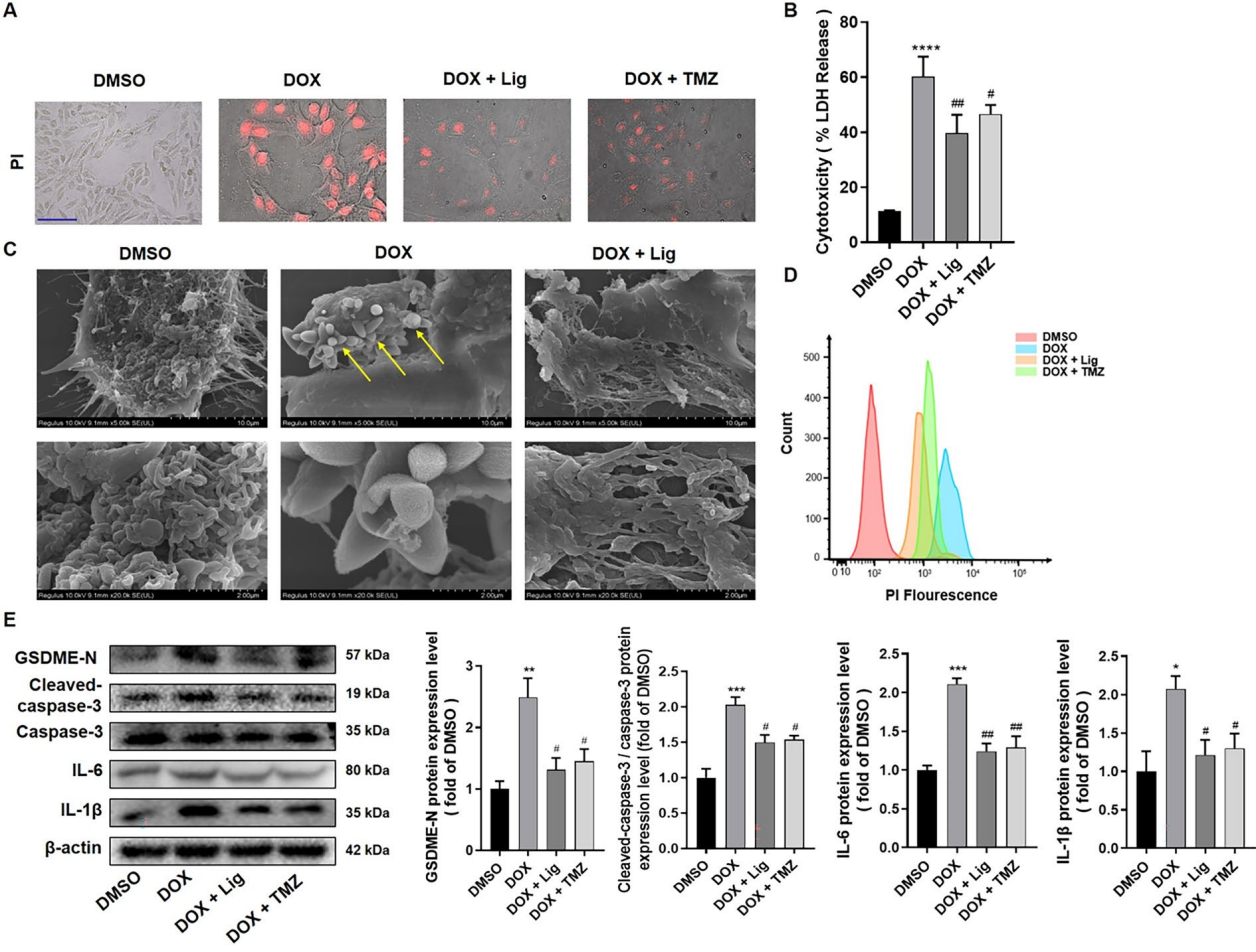
Lig treatment inhibits the caspase-3/GSDME signaling pathway in DOX-treated H9C2 cells. **A** Representative images of PI staining (n = 6). Scale bar represents 50 µm. **B** Effects of Lig on intracellular LDH level in H9C2 cells treated by DOX (n = 3). **C** Representative scanning electron microscopy (SEM) images showed the morphological changes of cultured H9C2 under different treatments (n = 3). **D** H9C2 cells stained with PI were detected by flow cytometry (n = 3). **E** The protein expression of GSDME-N, cleaved-caspase-3, caspase-3, IL-6, IL-1β in H9C2 cells (n = 3). Data are presented as the mean ± SD. *p < 0.05, **p < 0.01, ***p < 0.001, ****p < 0.0001 compared with control group; ^#^*p* < 0.05, ^##^*p* < 0.01 compared with model group

Mitochondrial dysfunction may be involved in caspase-3/GSDME-mediated cell pyroptosis. At the morphological level, we observed that H9C2 cells exhibited increasing pyroptotic membrane ballooning and took up PI red after DOX treatment. This effect was reversed with Lig and TMZ treatment, as observed in PI staining and flow experiments (Fig. 4A and D). Furthermore, pyroptosis leads to increased LDH release, and the results showed that Lig significantly decreased DOX-induced LDH release in H9C2 cells (Fig. 4B). Interestingly, these bubble-like protrusions, termed pyroptotic bodies, were previously observed in cells undergoing pyroptosis. As a distinct form of programmed cell death, pyroptosis can be distinguished from apoptosis by morphological features, where the latter shows cell shrinkage, cytoplasmic condensation, and the formation of apoptotic bodies with varying sizes around the nucleus [30]. It is noteworthy that pyroptosis is more prominently observed in cells upon treatment with DOX compared to Lig treatment (Fig. 4C). Subsequently, we tested the levels of pyroptosis-associated proteins, and the results indicated that the expressions of GSDME, Cleaved-caspase-3, IL-6, IL-1β and NF-κB proteins increased in DOX-treated H9C2 cells. However, these increases were significantly reversed after Lig treatment (Fig. 4E and Fig. S3C). These results demonstrate that Lig has the capability to inhibit pyroptosis in DOX-treated H9C2 cells.

### Activation of AMPK/SIRT3 signaling is required for Lig to reduce the mitochondrial respiration and pyroptosis in cardiomyocytes

**Fig. 5:**
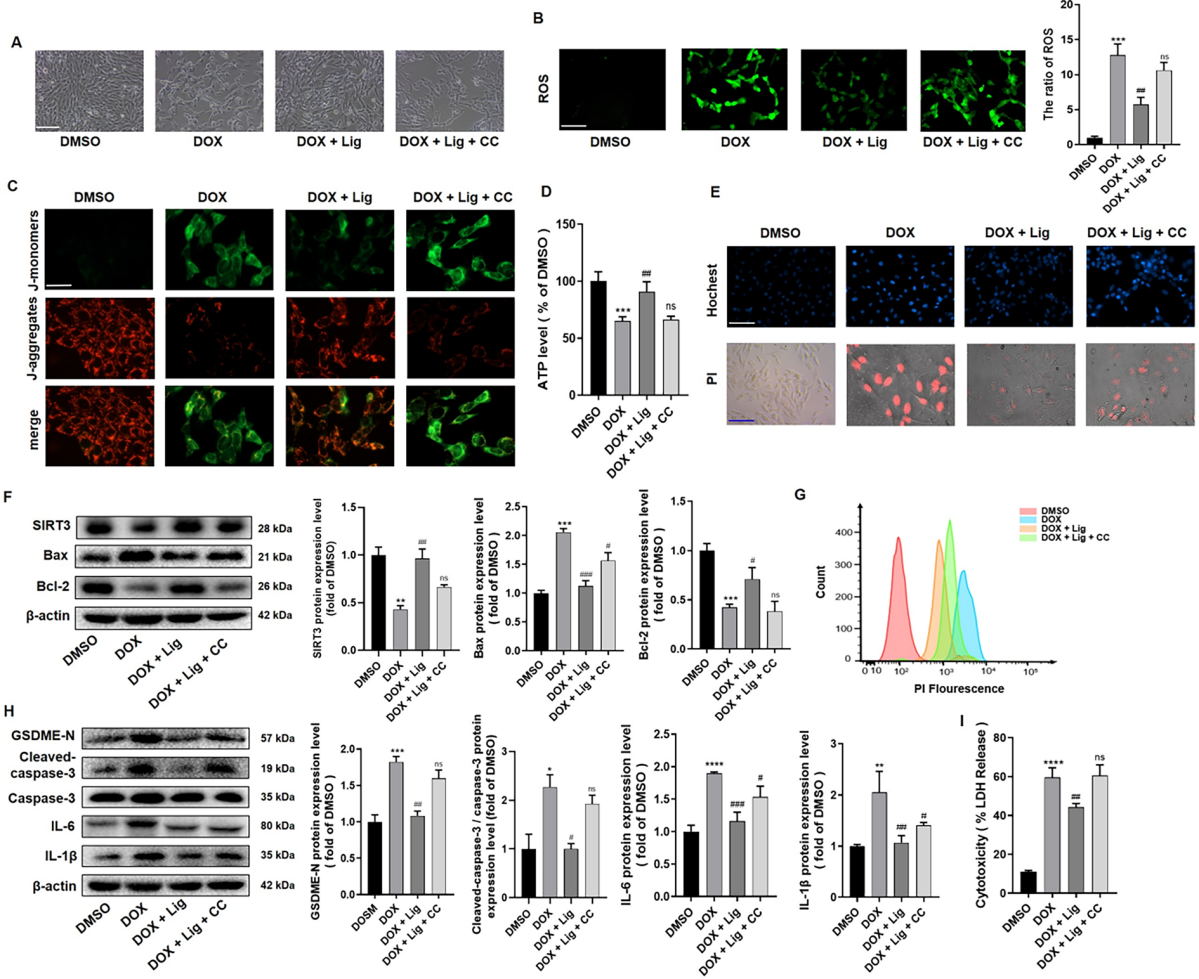
Activation of AMPK/SIRT3 signaling is required for Lig to reduce the mitochondrial respiration and pyroptosis in cardiomyocytes. **A** Morphology in H9C2 cells. Scale bar represents 100 µm (n = 6). **B** Intracellular ROS level in H9C2 cells (n = 4). Scale bar represents 50 µm. **C** MMP level was measured by JC-1 assay kit. Scale bar represents 25 µm (n = 6). **D** Effects of Lig and CC on intracellular ATP level in H9C2 cells (n = 3). **E** Representative images of Hochest and PI staining (n = 6). Scale bar represents 50 µm. **F** The expression of SIRT3, Bax and Bcl-2 in H9C2 cells treated by DOX (n = 3). **G** H9C2 cells stained with PI were detected by flow cytometry (n = 3). **H** The protein level of GSDME-N, cleaved-caspase-3, caspase-3, IL-6, IL-1β in H9C2 cells. (n = 3) **I** Intracellular LDH level in H9C2 cells treated by DOX (n = 3). Data are presented as the mean ± SD. *p < 0.05, **p < 0.01, ***p < 0.001, ****p < 0.0001 compared with control group; ^#^*p* < 0.05, ^##^*p* < 0.01 compared with model group. ns indicates no significance

To further elucidate the relationship between Lig and AMPK/SIRT3 signaling, we employed the AMPK inhibitor (CC) and specific AMPK-siRNA to assess cytotoxicity in DOX-induced H9C2 cells. Western blotting analysis confirmed the effective inhibition of siAMPK in H9C2 cells (Fig. S6A). Our findings revealed that CC treatment did not inhibit cell viability, but it significantly prevented the protective effects of Lig in DOX-treated H9C2 cells. This suggests that AMPK/SIRT3 signaling is essential for Lig to mitigate DOX-induced cytotoxicity in H9C2 cells (Fig. S3). Moreover, the cell morphology exhibited similar characteristics in Fig. 5A. As a crucial indicator of mitochondrial damage, DCFH-DA serves as a general ROS probe, highlighting the burst of ROS after stimulating cells with 3 μM DOX. However, this effect was mitigated by Lig treatment and then reversed with the AMPK inhibitor CC or siAMPK treatment (Fig. 5B and Fig S6B). The results of ROS by flow cytometry detection were consistent with the results of ROS fluorescence (Fig. S5A). ROS can induce modifications in related proteins in respiratory chain complexes and even interfere with DNA and RNA replication, affecting their structure and function at the transcriptional level. This cascade of events can lead to oxidative respiratory chain dysfunction and, ultimately, cellular energy metabolism disorders [31]. Subsequently, we utilized JC-1 to assess MMP, and observed that the green (monomeric) fluorescence was increased in the DOX treatment group compared to the Lig treatment group (Fig. 5C). Moreover, treatment with AMPK inhibitor CC or siAMPK reversed the effect of Lig and increased green fluorescence (Fig. 5C and Fig S6C). This result suggests that mitochondrial dysfunction may be a critical factor in the cell death processes induced by DOX, in which Lig reverses H9C2 cell death and AMPK plays an important role.

Furthermore, as MMP is crucial for ATP generation, we treated H9C2 cells with Lig and CC to investigate whether Lig would affect ATP levels. The results demonstrated that samples treated with Lig exhibited significantly higher ATP activity compared to the DOX group, indicating that Lig could enhance ATP release. However, this effect was diminished after AMPK inhibition (CC), suggesting that the improvement of mitochondrial function by Lig was attenuated by inhibiting AMPK (Fig. 5D). Mitochondria play a pivotal role in regulating apoptosis signals. Functional damage to mitochondria can result in an increase in MMP, leading to the leakage of internal substances from mitochondria and the activation of apoptosis [32]. Hoechst staining confirmed that the anti-apoptotic effect of Lig was compromised after combining Lig with CC, and PI staining indicated an increase in the DOX treatment group (Fig. 5E, Fig. S5B and S6D). Similarly, western blotting analysis revealed an increase in Bax expression and a decrease in Bcl-2 levels after AMPK inhibition, indicating that inhibiting AMPK weakened the anti-apoptotic effect of Lig (Fig. 5F and Fig. S6E). As an indicator of pyroptosis, increased LDH release was detected in H9C2 cells after AMPK inhibition, and the PI staining results were consistent with the above findings (Fig. 5G and I and Fig. S6D). When combined with CC, the inhibitory effect of Lig was mitigated, as evidenced by increased expression levels of GSDME-N and Cleaved-caspase-3 (Fig. 5H). Similarly, in combination with siAMPK, as with CC, expression levels of GSDME-N and Cleaved-caspase-3 increased significantly (Fig. S6F). Inhibition of AMPK activity using CC cotreatment counteracted Lig's anti-apoptotic effects by elevating the cell apoptotic index. These results underscore the involvement of AMPK activation in Lig's anti-apoptotic effect against DOX-induced cardiotoxicity.

### Knockdown of GSDME enhances the protective effect of Lig against DOX-induced H9C2 cell apoptosis

To validate the role of GSDME-mediated pyroptosis in the protective action of Lig, we employed specific GSDME-siRNA to knock down GSDME. Western blotting analysis confirmed the effective suppression of GSDME in H9C2 cells, achieving a transfection efficiency of approximately 25% (Fig. S7A). GSDME can be used as a key protein in apoptosis and pyrogenic transformation of cells. However, when GSDME is overexpressed, it will not only cause pyrogenic death of cells but also cause mitochondria to release pro-apoptotic factors to further cause apoptosis [33]. Following, combined treatment with siGSDME, the apoptosis induced by DOX in H9C2 cells was markedly reduced (Fig. S7B). Subsequently, we delved into the expression of apoptosis-related proteins in H9C2 cells. The western blotting assays unveiled that Lig treatment was linked to an upregulation in Bcl-2 expression and a downregulation in Bax expression compared to the DOX treatment group. Intriguingly, when combined with siGSDME treatment, the apoptosis of H9C2 cells was further diminished (Fig. S7C). The occurrence of pyroptosis leads to the release of a large number of inflammatory factors. Consequently, the expression levels of inflammation-related proteins IL-6 and IL-1β witnessed a further decrease in the Lig combined with siGSDME group compared to the Lig-alone group (Fig. S7C). Collectively, these findings suggest that GSDME knockdown may augment the protective efficacy of Lig against DOX-induced cytotoxicity in H9C2 cells.

### Effect of Lig treatment combined with AMPK inhibitors on cardiac toxicity, dysfunction, mitochondrial oxidative stress injury and pyroptosis on DOX-stimulated mice hearts

**Fig. 6:**
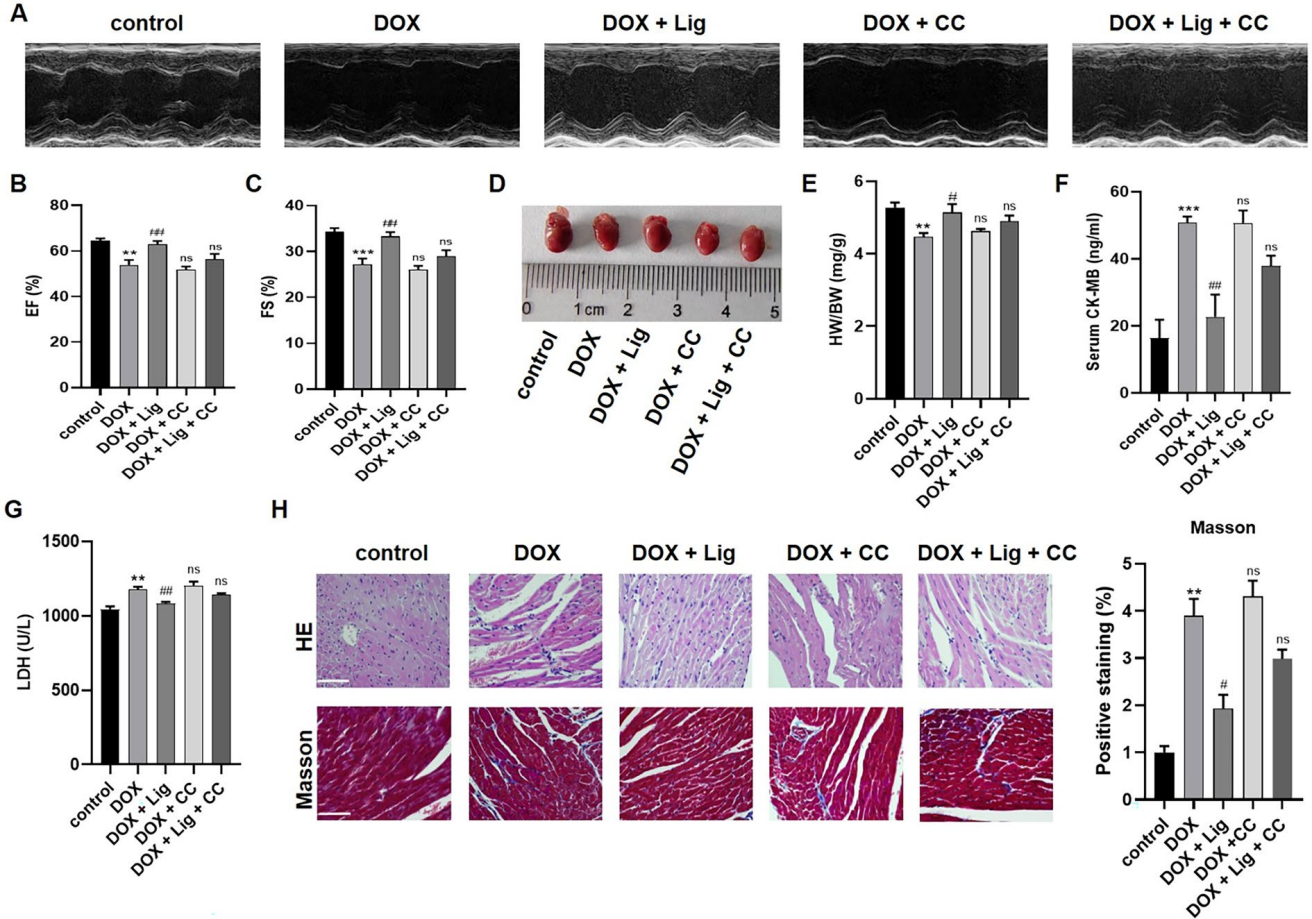
Effects of Lig combined with AMPK inhibitor on cardiac structure and function in DOX-induced mice. **A**–**C** The LVEF and LVFS were measured by echocardiography in each group (n = 6). **D**, **E** The gross heart pictures and the HW/BW ratio of mice (n = 10). **F**, **G** Effects of Lig and CC on serum levels of CK-MB and LDH in mice (n = 6). **H** HE and Masson staining images of heart tissues (n = 3). Data are presented as the mean ± SD. **p < 0.01, ***p < 0.001 compared with control group; ^#^*p* < 0.05, ^##^*p* < 0.01 compared with model group. ns indicates no significance

DOX has served as a pivotal component in chemotherapy for over five decades, forming the foundation of treatment protocols for various pediatric and adult cancers. However, its cardiotoxic effects have emerged as a growing and significant clinical challenge confronted by cardiologists [2, 3]. Previously, DOX-related mitochondrial dysfunction has become a likely pathological mechanism for DOX-induced cardiotoxicity, which is also a major reason ROS generation caused by DOX results in irreversible myocardial oxidative injury. In line with prior investigations, when the total cumulative dose of DOX reached 25 mg/kg, echocardiography demonstrated that CC nullified the beneficial impact of Lig on enhancing cardiac function in DOX-exposed mice (Fig. 6A–C). Combining Lig with CC led to a corresponding reduction in heart weight/body weight ratios (Fig. 6D and E). In addition, myocardial damage indexes CK-MB and LDH showed an increase after CC treatment, but there was no significant difference between CK-MB and Lig group. (Fig. 6F and G). This trend was also evident in HE and Masson tricolor staining, where CC annulled the positive effects of Lig, exacerbating inflammatory infiltration and collagen deposition in the myocardium of DOX-exposed mice (Fig. 6H). In summary, these findings underscored that Lig alleviated DOX-induced cardiac dysfunction, and when combined with AMPK inhibitor CC, CC partially reverses the protective effect of Lig, making Lig virtually ineffective in vivo.

**Fig. 7:**
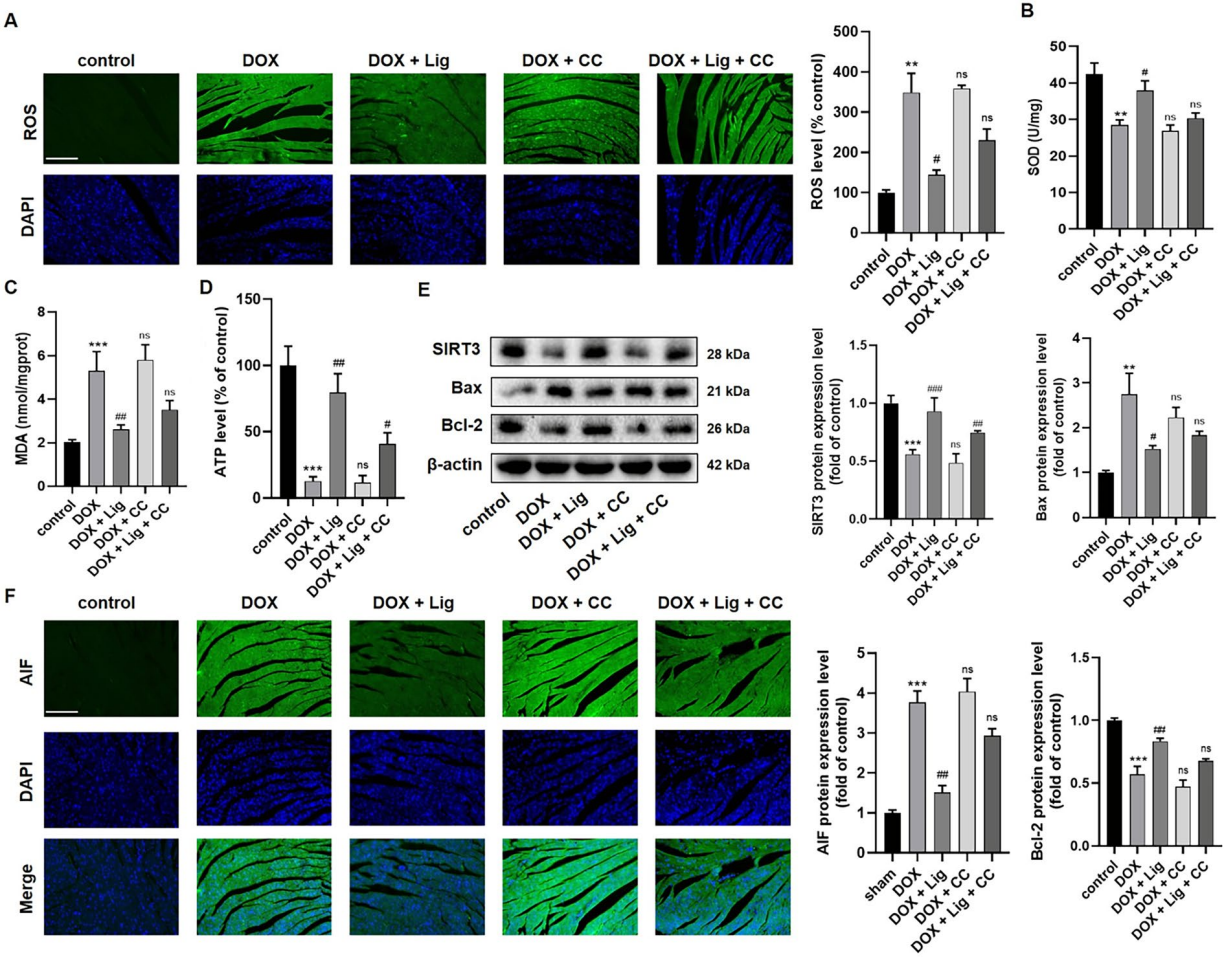
Effect of Lig treatment combined with AMPK inhibitors on cardiac mitochondrial oxidative stress injury and pyroptosis in mice. **A** The heart levels of ROS in mice caused by DOX (n = 3). **B**–**D** The heart levels of MDA, SOD and ATP in mice (n = 6). **E** The protein expression of SIRT3, Bax and Bcl-2 in mice treated by DOX (n = 3). **F** Immunofluorescence detects the levels of AIF on cardiac (n = 3). Data are presented as the mean ± SD. **p < 0.01, ***p < 0.001 compared with control group; ^#^*p* < 0.05, ^##^*p* < 0.01, ^###^*p* < 0.001 compared with model group. ns indicates no significance

Considering that mitochondrial oxidative stress is the primary contributor to DOX-induced cardiomyopathy, we investigated the oxidative stress parameters in myocardial tissue. The co-administration of Lig with CC significantly attenuated the antioxidative stress effect of Lig on the heart subjected to DOX stimulation. Compared to the Lig treatment group, there was an increase in ROS and MDA contents, a decrease in SOD activity, and a significant reduction in ATP content after treatment with Lig combined with CC (Fig. 7A–D). Additionally, the co-treatment with Lig and CC resulted in increased cardiomyocyte apoptosis, counteracting the anti-apoptotic effect of Lig (Fig. 7E). Tissue immunofluorescence of AIF revealed a significant increase in the combination treatment with CC (Fig. 7F). In Fig. s5A, it is evident that CC nullifies the effect of Lig on the reduction of GSDME-N and cleaved-caspase-3. Furthermore, inflammation-related factors were counteracted by CC, as evidenced by increased levels of IL-6 and IL-1β proteins, and the expression of IL-1β and IL-18 in the serum was consistent with these findings (Fig. s5B and C). Collectively, these results suggest that Lig can alleviate DOX-induced cardiotoxicity, reduce myocardial oxidative stress and apoptosis through the AMPK/SIRT3 signaling pathway, and inhibit the GSDME/caspase-3 pathway to mitigate DOX-induced pyroptosis of cardiomyocytes.

## Discussion

The cardiotoxic effects triggered by DOX have emerged as a significant clinical concern. Consequently, cardioprotectants are being explored as potential interventions to mitigate the cardiotoxicity induced by DOX [2, 34]. In this study, we illustrated that DOX treatment not only diminished the viability of H9C2 cells but also heightened cardiomyocyte apoptosis. However, co-treatment with Lig effectively reversed the myocardial damage, aligning with our previous research [20]. The findings indicate that Lig enhances DOX-induced cardiac function by elevating LVFS and LVEF in vivo. It has been reported that the accumulation of ROS induced by DOX can impede mitochondrial function by compromising DNA integrity and reducing mitochondrial membrane potential [35] which is the main reason for irreversible myocardial oxidative damage induced by DOX [5]. In this study, the levels of ROS and MDA were markedly elevated, and SOD activity decreased in the myocardial tissue of mice exposed to DOX. The combined therapy with Lig effectively reversed these detrimental effects. Furthermore, DOX triggered the pyroptosis of cardiomyocytes, and Lig treatment ameliorated this phenomenon, as evidenced by reduced expression of GSDME-N and cleaved-caspase-3 protein levels. Notably, with the increase in AMPK protein levels, myocardial oxidative stress, injury, and pyroptosis were alleviated, suggesting that Lig mitigated DOX-induced cardiotoxicity by upregulating AMPK expression.

DOX-induced cardiomyopathy involves a complex interplay of various cell death mechanisms. Among these, the induction of cardiomyocyte apoptosis is recognized as a significant contributor to the pathogenesis of DOX-induced cardiotoxicity [24]. and the main cause of cardiac tissue loss and cardiac insufficiency during mitochondria-dependent intrinsic apoptosis [36]. Studies have reported that Lig has a myocardial protective effect on apoptosis under various pathogenic conditions [19, 20]. To assess DOX-induced cardiomyocyte apoptosis, we conducted an immunofluorescence assay and examined apoptotic protein levels. The findings revealed that Lig treatment effectively suppressed DOX-induced apoptosis by decreasing the levels of pro-apoptotic proteins (Bax and AIF) and increasing the levels of anti-apoptotic Bcl-2 protein. Notably, the inhibition of AMPK reversed the protective effect of Lig on the myocardium, implicating AMPK in the anti-apoptotic protective mechanism of Lig against cardiotoxicity.

Pyroptosis, a recently identified form of programmed cell death, is characterized by the formation of cell membrane pores, cell swelling, and eventual cell membrane rupture. Its distinctive features include the ultimate expression and release of inflammatory factors [33, 37]. Excessive inflammation has been identified as a trigger for the activation of pyroptosis. Current research indicates that caspase-3 activation can lead to GSDME-dependent pyroptosis. High expression of GSDME in the presence of activated caspase-3 can induce GSDME-dependent pyroptosis. Conversely, when GSDME expression is low, caspase-3 activation may primarily induce apoptosis [38]. As a member of the Gasdermin protein family, GSDME is composed of cytotoxic GSDME-N and GSDME-C. When cells are stimulated by external therapeutic factors or death signals, the activation of caspase-3 triggers the cleavage of GSDME into GSDME-N fragments. This process induces the formation of pyroptosis, leading to the release of inflammatory cytokines such as IL-1β and IL-18 [33, 39]. Both in vivo and in vitro, we confirmed the results of these experiments, demonstrating that DOX can promote the occurrence of pyroptosis of cardiomyocytes and aggravate the inflammatory response. After the addition of GSDME siRNA in vitro, these phenomena were inhibited, and these adverse reactions were also reversed under Lig treatment.

**Fig. 8:**
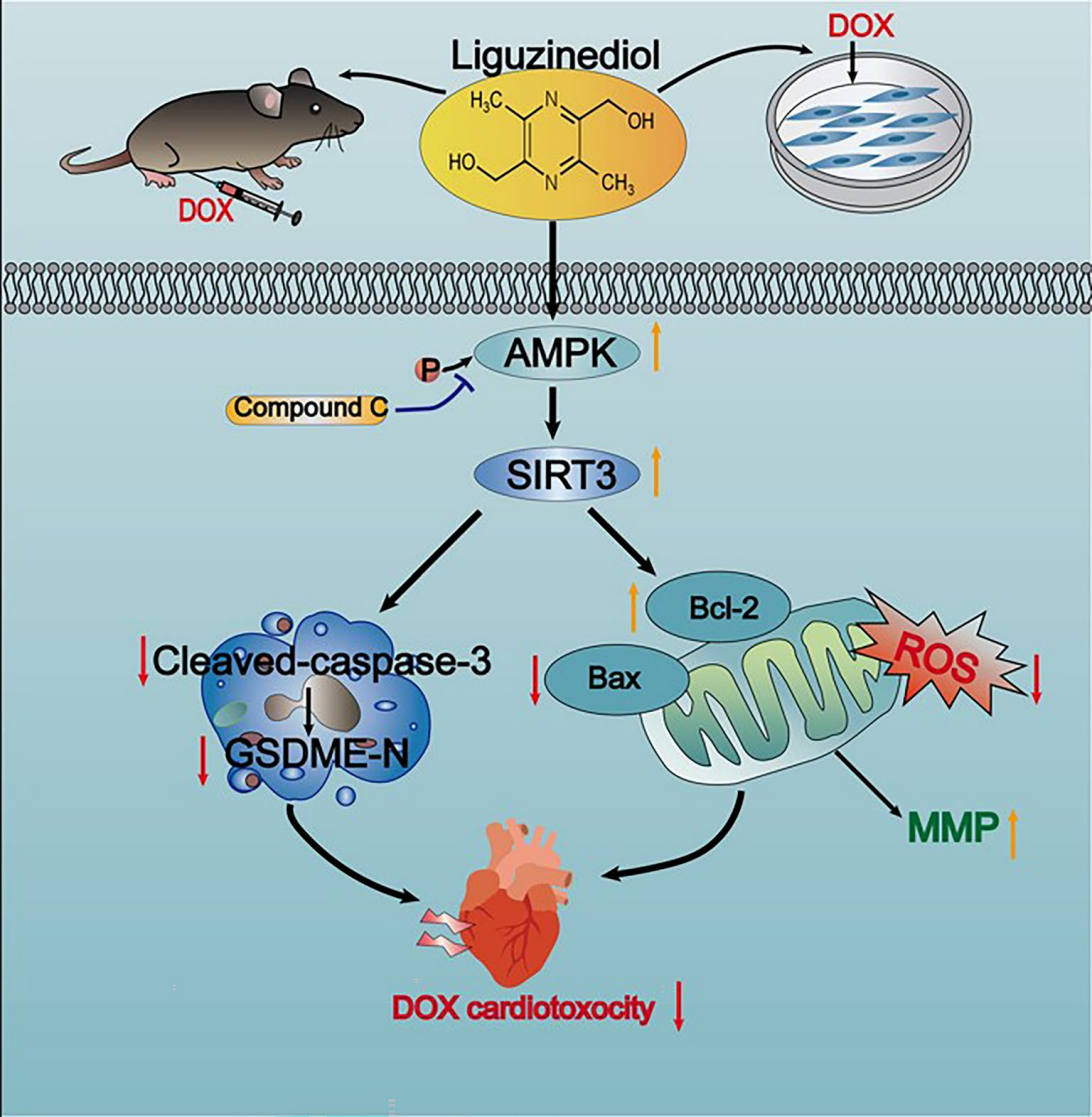
Graphical abstract of how Lig ameliorates DOX-induced cardiotoxicity and potentiates the metabolic remodeling by activating the AMPK/SIRT3 pathway and represses Caspase-3/GSDME-mediated pyroptosis

AMPK is an evolutionarily conserved cellular energy manager that controls energy homeostasis and signaling in the heart [38]. Phosphorylation at Thr172 significantly increases AMPK activity to maintain energy balance [40]. In contrast to cardiac diseases such as heart failure and myocardial ischemia [41, 42]. Myocardial AMPK activation was significantly impaired in both acute and chronic DOX cardiac toxicity [24]. In this study, we observed that Lig therapy significantly reversed DOX-induced downregulation of AMPK phosphorylation in H9C2 and mouse heart tissue. In addition, the application of AMPK inhibitor CC significantly inhibited the protection of Lig against cardiotoxicity in DOX by promoting cardiac dysfunction, myocardial tissue structure change, apoptosis, and oxidative stress. SIRT is a member of the NAD-dependent enzyme family and is a well-known long-lived protein [43]. SIRT3 localization with mitochondria or nucleus [15]. More and more evidence confirms the regulation of SIRT3 by AMPK [12, 44]. In this study, we found that AMPK inhibitor CC inhibited SIRT3 upregulation in Lig-treated DOX-stimulated cardiomyocytes. These results suggest the importance of AMPK/SIRT3 signaling pathway in the treatment of DOX-induced cardiotoxicity, and that Lig may enhance its signaling pathway to ameliorate DOX cardiotoxicity (Fig. 8).

## Conclusions

Our findings suggest that Lig can effectively alleviate DOX-induced cardiomyocyte apoptosis and oxidative stress damage. Notably, Lig improves DOX-induced cardiotoxicity by stimulating AMPK/SIRT3 signaling and inhibiting the Caspase-3/GSDME pyrogenic signaling pathway, which represents a novel mechanism of action. These findings reveal previously unappreciated therapeutic targets for DOX-induced cardiotoxicity and hold promise for Lig as a new approach to the treatment of heart disease.

### Supplementary Information

Supplementary Material 1.

## Data Availability

Please contact author for data requests.

## Abbreviations

Lig: Liguzinediol
DOX: Doxorubicin
AMPK: AMP-activated protein kinase
p-AMPK: Phosphorylated- AMP-activated protein kinase
SIRT3: Sirtuin 3
GSDME-N: Gasdermin E N-terminal fragment
ROS: Reactive oxygen species
MDA: Malondialdehyde
SOD: Superoxide dismutase
CK-MB: Creatine kinas-MB
LDH: Lactate dehydrogenase
CC: AMPK inhibitor compound C
IL-1β: Interleukin-1β
IL-6: Interleukin-6
TMZ: Trimetazidine
CCK-8: Cell Counting Kit-8
ATP: Adenosine triphosphate
LVEF: Left ventricular ejection fraction
FS: Fraction shortening
PI: Propidium iodide
MMP: Mitochondrial membrane potential
HE: Hematoxylin and eosin
LVSP: Left ventricular systolic pressure
LVEDP: Left ventricular end-diastolic pressure
NO: Nitric oxide
FFA: Free fatty acids
