# Supplementary material for: Liguzinediol potentiates the metabolic remodeling by activating the AMPK/SIRT3 pathway and represses Caspase-3/GSDME-mediated pyroptosis to ameliorate cardiotoxicity
Source: Chin Med. 2024 Jun 14;19:85. doi: 10.1186/s13020-024-00955-5 (PMC11179277; doi:10.1186/s13020-024-00955-5)
Supplement: Supplementary file 1 — Supplementary Material 1. [file 13020_2024_955_MOESM1_ESM.docx]

**Liguzinediol potentiates the metabolic remodeling by activating the AMPK/SIRT3 pathway and represses Caspase-3/GSDME-mediated pyroptosis to ameliorate cardiotoxicity**

Weijie Zhu^a#^, Naqi Lian^a#^, Jia Wang^a^, Fengming Zhao^a^, Bowen Liu^a^, Jiaxing Sheng^a^, Chenyan Zhang^a^, Xuan Zhou^b^, Wenbai Gao^a^, Chen Xie^c^, Haoyu Gu^a^, Yuxin Zhang^a^, Mianli Bian^a^*, Miao Jiang^a^*, Yu Li^a^*

^a^ *School of Medicine,* *Nanjing University of Chinese Medicine, Nanjing 210023, China*

*^b^ School of Senior Care Services and Management, Nanjing University of Chinese Medicine, Nanjing 210023, China*

*^c^ College of Acupuncture and Massage health and rehabilitation, Nanjing University of Chinese Medicine, Nanjing 210023, China*

#The first two authors contributed equally to this work.

***Correspondence**

Dr. Mianli Bian, E-mail: [bianmianli@njucm.edu.cn](mailto:bianmianli@njucm.edu.cn).

Dr. Miao Jiang, E-mail: jiangmiao820701@163.com.

Dr. Yu Li, E-mail: liyu@njucm.edu.cn.

**
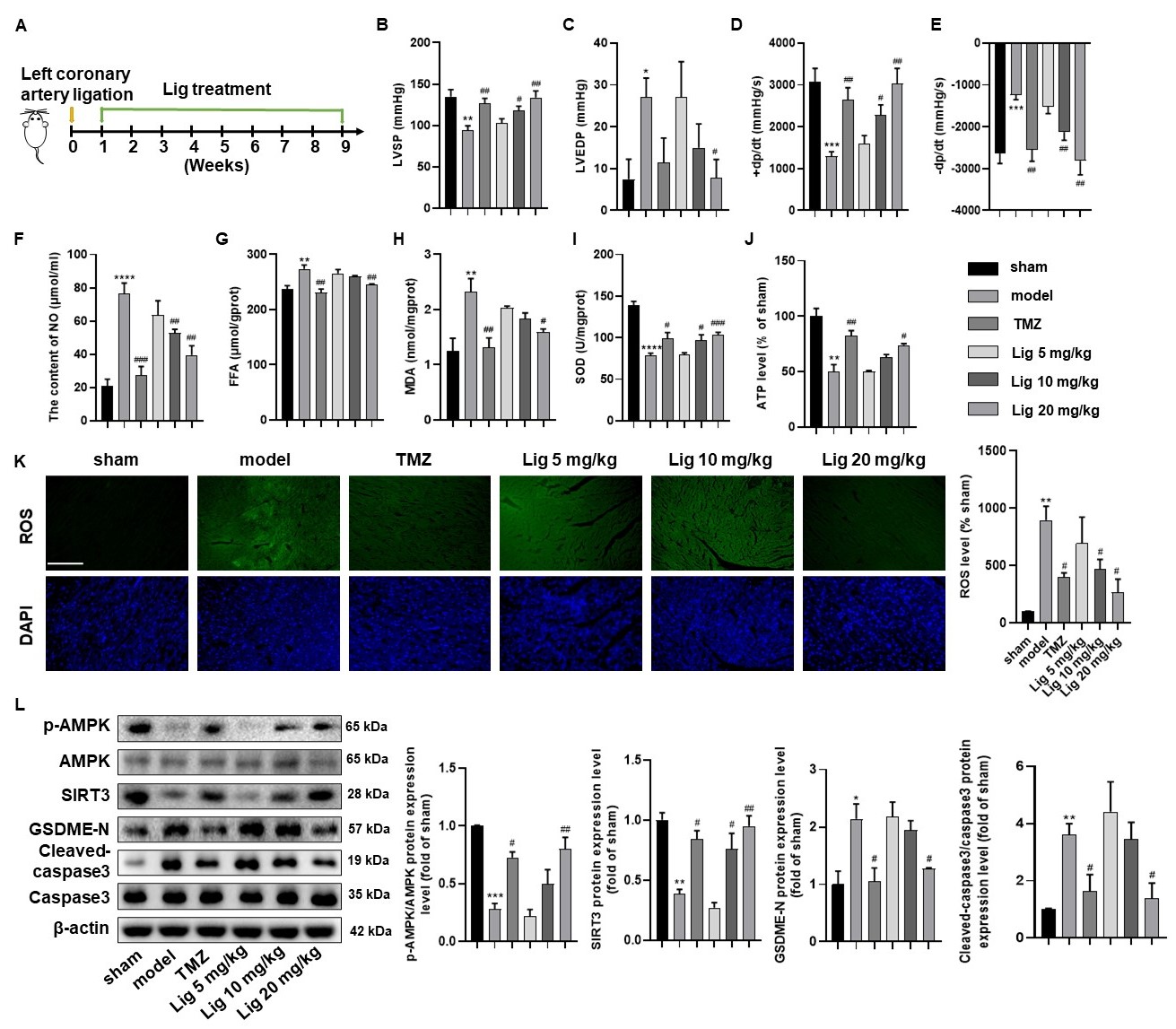
**

**Fig. S1. Lig improves cardiac function in rats, activates AMPK/SIRT3 pathway, reduces caspase-3/GSDME-N mediated pyroptosis, and alleviates heart failure.** (A) Schematic diagram of coronary ligation and Lig treatment. (B-E) Left ventricular function was measured by hemodynamics, including LVSP, LVEDP, +dp/dt max and -dp/dt max (n = 6). (F-J) Effects of Lig on NO, FFA, MDA, SOD and ATP in rats with heart failure (n = 4). (K) Effects of Lig on heart levels of ROS in rats with heart failure (n = 3) (L) Protein expression of p-AMPK, AMPK, SIRT3, GSDME-N, Cleaved-caspase-3, and caspase-3 in rats with heart failure (n = 3). Data are presented as the mean ± SD. **P* < 0.05, ***p* < 0.01, ****p* < 0.001, *****p* < 0.0001 compared with sham control group; ^#^*p* < 0.05, ^##^*p* < 0.01, ^###^*p* < 0.001 compared with model group.

**
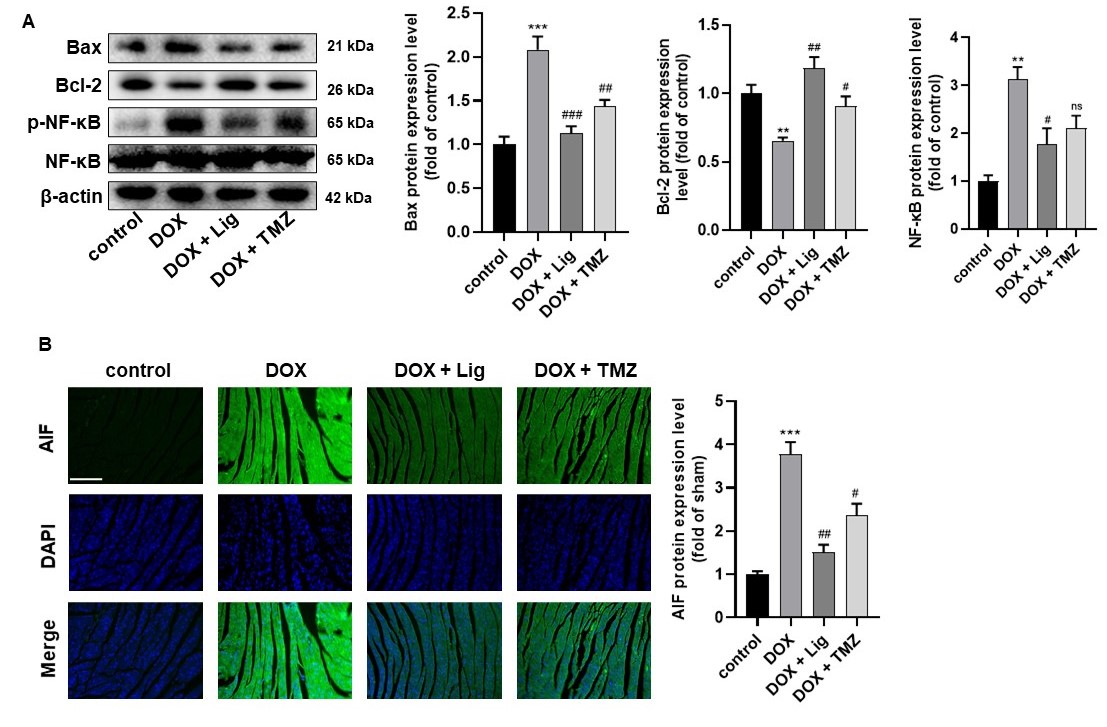
**

**Fig. S2. Lig inhibits cardiac apoptosis induced by DOX in mice.** (A) Effects of Lig on the expression levels of Bax, Bcl-2, p-NF-κB and NF-κB in mice treated by DOX (n = 3). (B) AIF expression in mice treated by DOX (n = 3). Data are presented as the mean ± SD. ***p* < 0.01, ****p* < 0.001 compared with control group; ^#^*p* < 0.05, ^##^*p* < 0.01, ^###^*p* < 0.001 compared with DOX model group. ns indicates no significance.

**
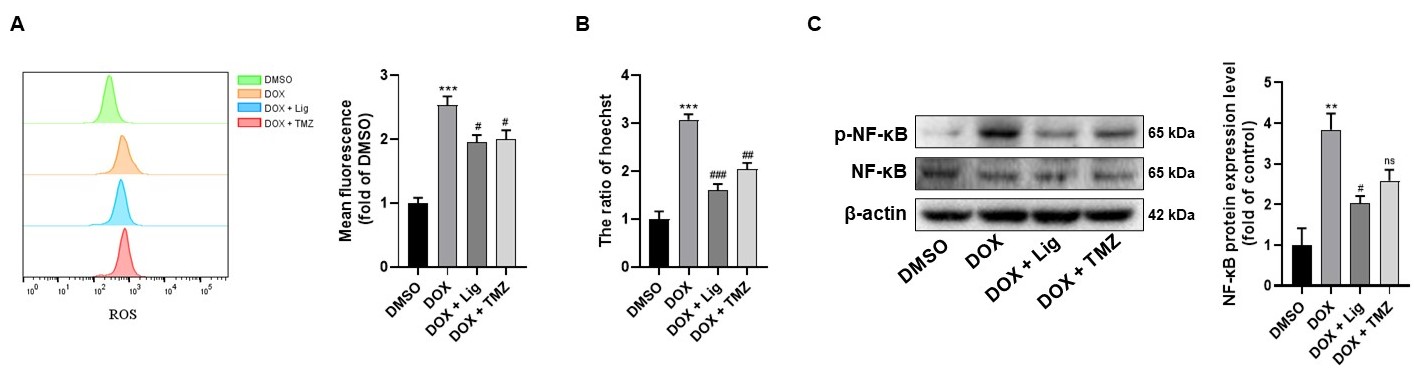
**

**Fig. S3. Lig decreased DOX-induced H9C2 cells mitochondrial oxidative stress, and cell apoptosis by improving AMPK/SIRT3 signaling.** (A) The ROS content was detected by flow cytometry (n = 3). (B) Quantification of Hochest staining (n = 6). (C) Effects of Lig on the expression levels of p-NF-κB and NF-κB in mice treated by DOX (n = 3). Data are presented as the mean ± SD. ***p* < 0.01, ****p* < 0.001 compared with control group; ^#^*p* < 0.05, ^##^*p* < 0.01, ^###^*p* < 0.001 compared with DOX model group.

**
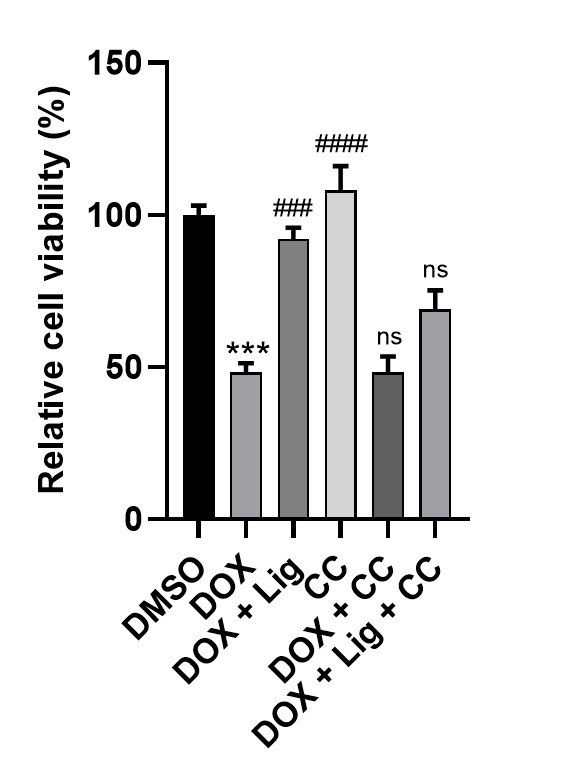
**

**Fig. S4. The activity of H9C2 cells under the intervention of AMPK inhibitor CC.** Cell viability in H9C2 cells (n = 5). Data are presented as the mean ± SD. ****p* < 0.001 compared with DMSO control group; ^###^*p* < 0.001, ^####^*p* < 0.0001 compared with DOX model group. ns indicates no significance.

**
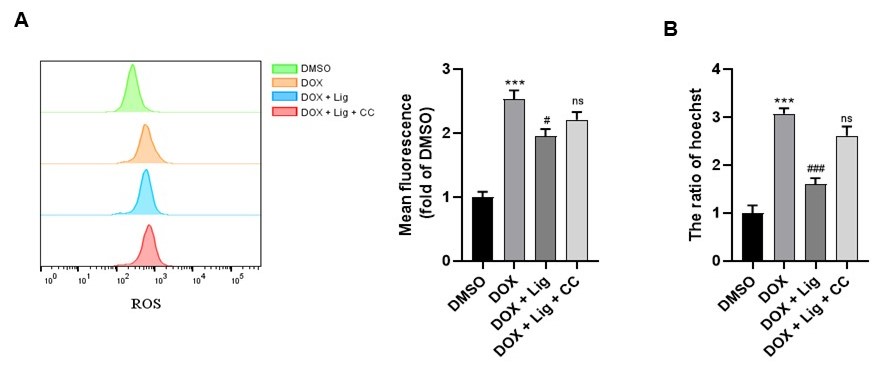
**

**Fig. S5. Activation of AMPK/SIRT3 signaling is required for Lig to reduce the oxidative stress and apoptosis in cardiomyocytes.** (A) The ROS content was detected by flow cytometry (n = 3). (B) Quantification of Hochest staining (n = 6). Data are presented as the mean ± SD. ****p* < 0.001 compared with control group; ^#^*p* < 0.05, ^###^*p* < 0.001 compared with DOX model group. ns indicates no significance.

**
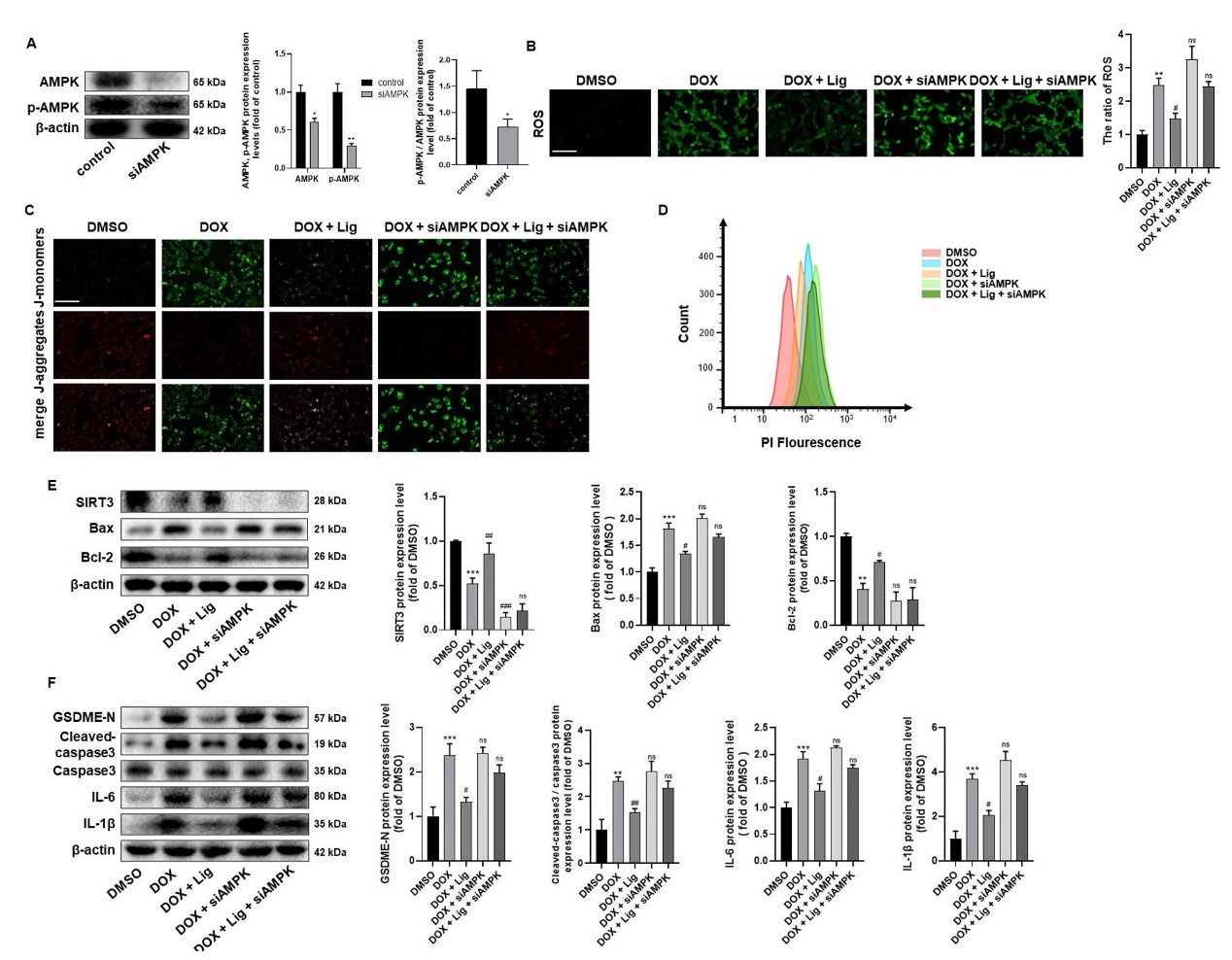
**

**Fig. S6. Activation of AMPK/SIRT3 signaling is required for Lig to reduce the mitochondrial respiration and pyroptosis in cardiomyocytes.** (A) Effects of siAMPK on the expression levels of p-AMPK and AMPK in H9C2 cells (n = 3). (B) Intracellular ROS level in H9C2 cells (n = 4). Scale bar represents 50 µm. (C) MMP level was measured by JC-1 assay kit. Scale bar represents 50 µm (n = 3). (D) H9C2 cells stained with PI were detected by flow cytometry (n = 3). (E) The expression of SIRT3, Bax and Bcl-2 in H9C2 cells treated by DOX (n = 3). (F) The protein level of GSDME-N, cleaved-caspase-3, caspase-3, IL-6, IL-1β in H9C2 cells. (n = 3) Data are presented as the mean ± SD. **p* < 0.05, ***p* < 0.01, ****p* < 0.001, ****p* < 0.0001 compared with control group; ^#^*p* < 0.05, ^##^*p* < 0.01 compared with model group. ns indicates no significance.

**
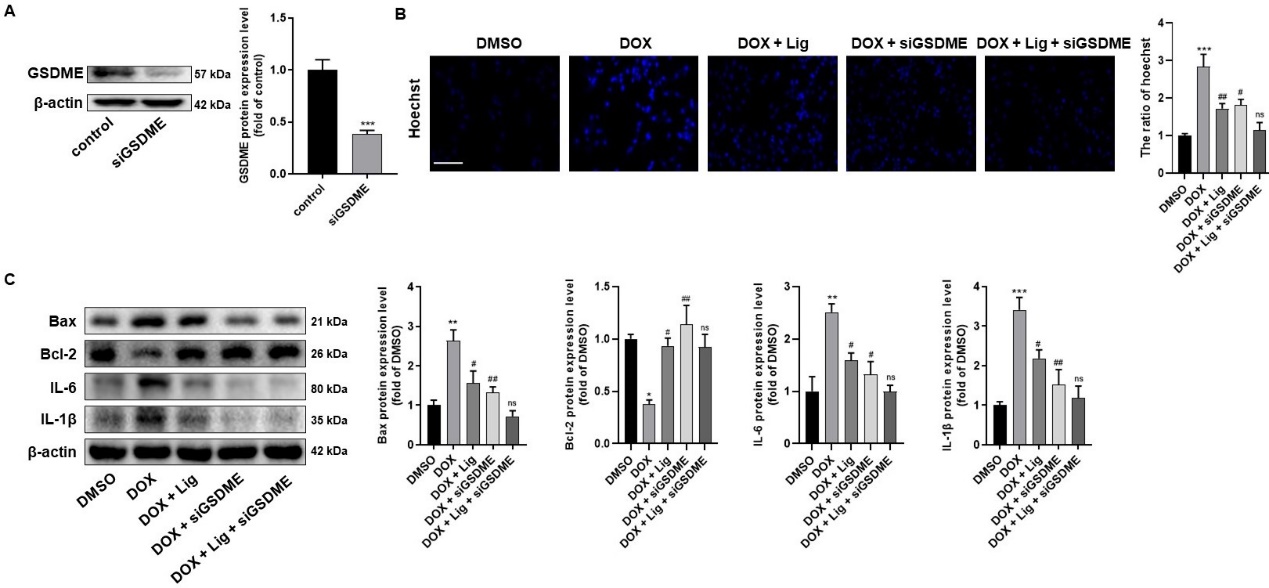
**

**Fig. S7. Knockdown of GSDME enhances the protective effect of Lig against DOX-induced H9C2 cytotoxicity.** (A) Effects of siGSDME on the expression levels of GSDME in H9C2 cells (n = 3). (B) Representative images and quantified results of Hoechst staining. Scale bar represents 50 µm (n = 5). (C) Effects of siGSDME and Lig on the expression levels of Bax, Bcl-2, IL-6 and IL-1β in DOX treated H9C2 cells (n = 3). Data are presented as the mean ± SD. **p* < 0.05, ***p* < 0.01, ****p* < 0.001 compared with DMSO control group; ^#^*p* < 0.05, ^##^*p* < 0.01 compared with DOX model group. ns indicates no significance.

**
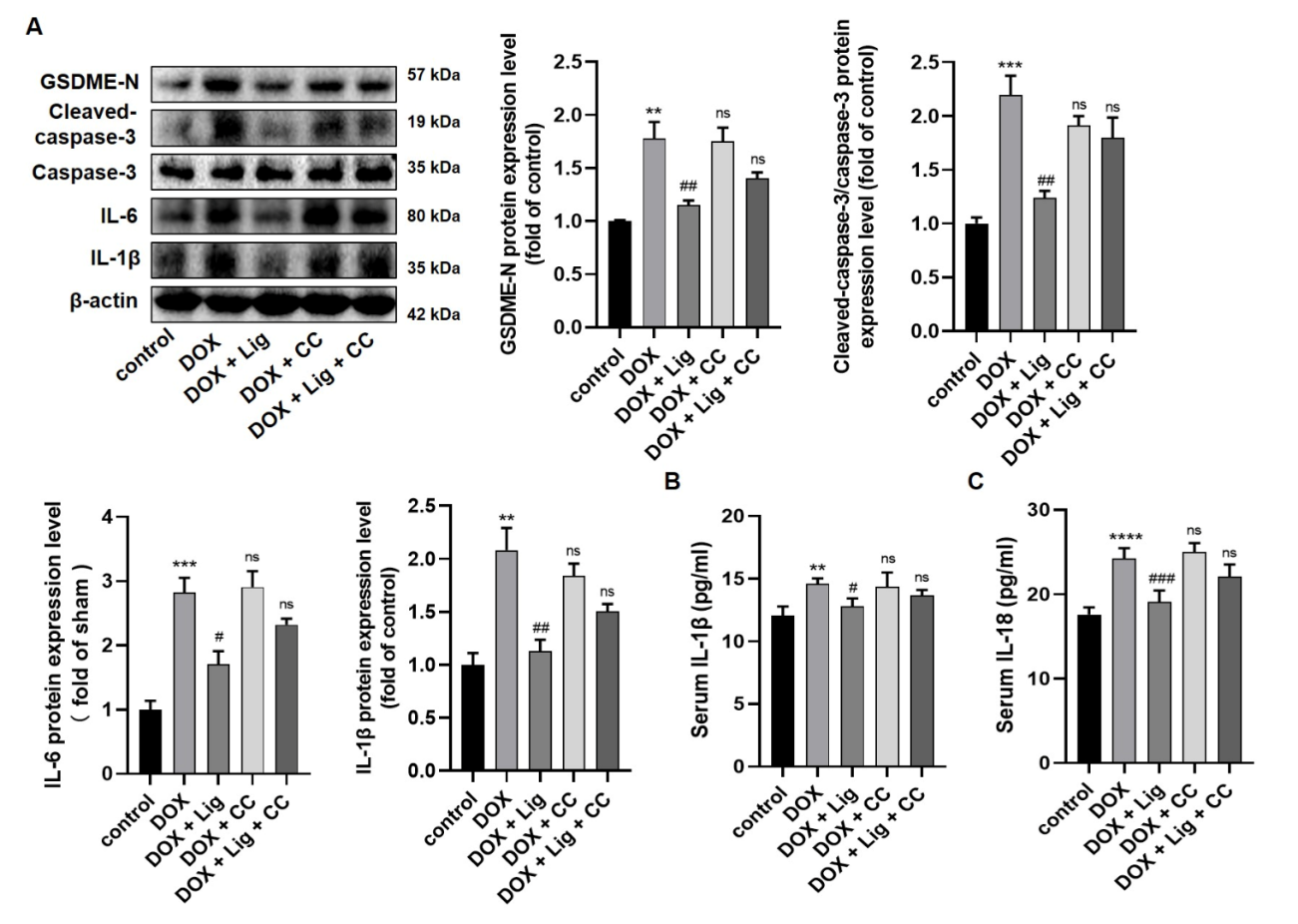
**

**Fig. S8. Effect of Lig combined with CC on DOX stimulation of caspase-3/GSDME pathway in mouse heart.** (A) Effects of Lig combined with CC on the expression levels of GSDME, cleaved-caspase-3, caspase-3, IL-6 and IL-1β in mice treated by DOX (n = 3). (B-C) Effects of Lig and CC on serum levels of IL-1β and IL-18 in mice caused by DOX. Data are presented as the mean ± SD. ***p* < 0.01, ****p* < 0.001, *****p* < 0.0001 compared with control group; ^#^*p* < 0.05, ^##^*p* < 0.01, ^###^*p* < 0.001 compared with DOX model group. ns indicates no significance.
